# MID-FTIR-PLS Chemometric Analysis of Cr(VI) from Aqueous Solutions Using a Polymer Inclusion Membrane-Based Sensor

**DOI:** 10.3390/membranes13080740

**Published:** 2023-08-18

**Authors:** Armando Martínez de la Peña, Eduardo Rodríguez de San Miguel, Josefina de Gyves

**Affiliations:** Departamento de Química Analítica, Facultad de Química, Universidad Nacional Autónoma de México (UNAM), Ciudad Universitaria, Mexico City 04510, Mexico; amdlp_1986@hotmail.com (A.M.d.l.P.); degyves@unam.mx (J.d.G.)

**Keywords:** chromium(VI), polymer inclusion membrane, optode, FTIR, quantitative analysis

## Abstract

A partial least squares (PLS) quantitative chemometric method based on the analysis of the mid-Fourier transform infrared spectroscopy (MID-FTIR) spectrum of polymer inclusion membranes (PIMs) used for the extraction of Cr(VI) from aqueous media is developed. The system previously optimized considering the variables membrane composition, extraction time, and pH, is characterized in terms of its adsorption isotherm, distribution coefficient, extraction percent, and enrichment factor. A Langmuir-type adsorption behavior with *K_L_* = 2199 cm^3^/mmol, *q_max_* = 0.188 mmol/g, and 0 < *R_L_* < 1 indicates that metal adsorption is favorable. The characterization of the extraction reaction is performed as well, showing a 1:1 Cr(VI):Aliquat 336 ratio, in agreement with solvent extraction data. The principal component analysis (PCA) of the PIMs reveals a complex pattern, which is satisfactorily simplified and related to Cr(VI) concentrations through the use of a variable selection method (iPLS) in which the bands in the ranges 3451–3500 cm^−1^ and 3751–3800 cm^−1^ are chosen. The final PLS model, including the 100 wavelengths selected by iPLS and 10 latent variables, shows excellent parameter values with root mean square error of calibration (*RMSEC*) of 3.73115, root mean square error of cross-validation (*RMSECV*) of 6.82685, bias of −1.91847 × 10^−13^, cross-validation (CV) bias of 0.185947, R^2^ Cal of 0.98145, R^2^ CV of 0.940902, recovery% of 104.02 ± 4.12 (α = 0.05), sensitivity% of 0.001547 ppb, analytical sensitivity (γ) of 3.8 ppb, γ^−1^: 0.6 ppb^−1^, selectivity of 0.0155, linear range of 5.8–100 ppb, limit of detection (LD) of 1.9 ppb, and limit of quantitation (LQ) of 5.8 ppb. The developed PIM sensor is easy to implement as it requires few manipulations and a reduced number of chemical compounds in comparison to other similar reported systems.

## 1. Introduction

Due to the extensive and rapid development, environmental pollution has increased to alarming levels in nearby industrialized areas, which are generally contaminated with heavy metals. Among many others, hexavalent chromium, Cr(VI), is one of the most serious concerns, as there is credible evidence that through the water medium, it is a major contributor to the global burden of cancer in humans [1]. Chromium compounds are mainly used in industrial activities, such as corrosion control, oxidation process, leather tanning, electroplating, metallurgy, cement, textile dyeing, papermaking, inks, paints and pigments, and photographic industry [2]. Due to this wide use, there are locations where chromium compounds have been released to the environment via leakage, poor storage, or improper industrial disposal practices, so chromium pollution of waters and groundwaters represents a serious environmental concern. The USEPA regulates total chromium in drinking water and has set a Maximum Contaminant Level (MCL) of 0.1 mg/dm^3^ [3]. The World Health Organization (WHO) guideline is 0.05 mg/dm^3^ for total chromium [4]. Although this reference value has been questioned, as a practical measure, it has been retained as a provisional guideline value until additional information becomes available and chromium can be re-evaluated [4]. Chromium discharge limits in water are regulated on a national scale and often vary depending on the different types of industry or receiving water bodies (marine water, lake, river, or sewer system) [5]. The maximum discharge limit to the aquatic environment in the EU is 1 and 5 mg/dm^3^ for Cr(VI) and Cr_total_, respectively [6].

There are currently several technologies for the remediation [5] and detection [7] of Cr(VI) from aqueous samples. As this species is usually present at low concentration levels, it is measured by atomic absorption and emission methods, providing high precision and sensitivity measurements. The selection of these techniques will depend largely on the sensitivity, reproducibility, detection and quantification limits, and simplicity of the method. However, environmental scientists are increasingly in need of measurement closer to the sample, in situations outside the laboratory, which restricts the use of such analytical techniques. Alternatively, UV/VIS, FTIR, Raman, and NIR portable spectrometers [8,9] are available to meet the growing need driven by the general trend in analytical instrumentation toward smaller size, improved reliability, and greater ease of operation. In such a context, the use of ion-selective optodes (ISOs), ionophore-based optical chemical sensors, represents an excellent alternative for the portable determination of ions [10]. In this regard, several systems for Cr(VI) monitoring using optical sensors have been developed in recent years [11,12,13,14].

On the other hand, besides being used for the removal and transport of metals [15,16], polymer inclusion membranes (PIMs) have also been employed for the detection of many chemical species [17]. These membranes are composed of a polymeric matrix, which serves as support of an encapsulated extractant (carrier) that is responsible for binding with the target analyte at the source solution/membrane interface and transporting it across the membrane, which can contain a plasticizer to improve mobility. In the case of Cr(IV), Aliquat 336 (methyltrioctylammonium chloride) has proved to be an effective and selective extractant in solvent extraction (SX) [18,19], supported liquid membranes (SLMs) [20], solvent-impregnated resins (SIR) [21,22], and PIM [20,23,24,25,26,27,28,29] systems. 

In previous work, the capabilities of a PIM sensor to perform cadmium(II) determination in aqueous solutions by in-situ visible (VIS) and mid-Fourier transform infrared spectroscopy (MID-FTIR) analyses of the polymeric films using a partial least squares (PLS) chemometric approach were demonstrated [30]. One major advantage of the developed MID-FTIR-PLS PIM-based method was that it did not require the presence in the membrane of a chemical reagent with special properties, either a chromophore species that can complex the metal ion, i.e., acting as an ionophore [14,31], or a mixture of an ionophore and a chromophore in the same PIM [32], or a fluorescent reagent [33]. However, to extend the potentiality of the methodology to low analyte concentration ranges, a careful selection of the dielectric nature of the medium and the dipole moment of the bond associated with IR vibrations of the extracted complex was suggested [30], so that an increase in the band intensities could be achieved, i.e., an augment in the magnitude of the analytical signal, improving the detection capabilities of these sensors. 

Taking advantage of the high dielectric constant values observed in PIMs with Aliquat 336 plasticized with 2-nitrophenyl octyl ether (NPOE) [34] and the excellent transport properties of Aliquat 336 for Cr(VI) in PIMs [20,23,24,25,26,27,28,29], in this work, such membranes were employed for MID-FTIR-PLS analysis of Cr(VI) from aqueous media. Once the system had been characterized and optimized, the calibration performance and the figures of merit (FOM) of the method were determined to show the potentiality of the proposed methodology.

## 2. Materials and Methods

### 2.1. Reagents and Apparatus

PIMs were prepared using cellulose triacetate (CTA, Honeywell Fluka, Charlotte, NC, USA) as polymer support, 2-nitrophenyl octyl ether (NPOE, ≥99.0% Honeywell Fluka) as a plasticizer, Aliquat 336 (≥97%, methyltrioctylammonium chloride, Sigma-Aldrich, Chem. Co., St. Louis, MO, USA) as an extracting agent, and dichloromethane (Merck, Kenilworth, NJ, USA) as a casting solvent. Working Cr(VI) solutions were prepared by dissolving the corresponding amounts of (NH_4_)_2_Cr_2_O_7_ (≥99.5%, Sigma-Aldrich) in deionized water (18 MΩ cm, MilliQ, Merck Millipore, Burlington, MA, USA). A 1000 mg/dm^3^ Sigma-Aldrich AAS standard solution (≥99.5%, Sigma-Aldrich, 1 g/dm^3^ Cr in 2% nitric acid, prepared with high purity (NH_4_)_2_Cr_2_O_7_, HNO_3_ and water) was diluted using deionized water for the preparation of the standards for flame atomic absorption spectrometry (FAAS) determinations. Tris (hydroxymethyl)aminomethane (TRIS, 99.8% Sigma-Aldrich), sodium acetate (99% Sigma-Aldrich), acetic acid (99.7% Sigma-Aldrich), hydrochloric acid (37% Sigma-Aldrich), and 2-(N-morpholino) ethanesulfonic acid (MES, 99.5% Sigma-Aldrich) were employed to assess the effect of pH on chromium extraction. 

A model 75Wrist ActionTM shaker (Burrell Scientific Inc., Pittsburgh, PA, USA) with 10 speeds and a Metrohm 620 pH-meter (Herisau, Switzerland) were used. A 3100-flame atomic absorption spectrometer (Perkin Elmer Waltham, MA, USA) and a Spectrum GX IR spectrometer (Perkin Elmer) were employed to measure metal ion contents and obtain the IR spectra, respectively. A Fowler IP54 micrometer (Fowler High Precision, Newton, MA, USA) was used for measuring PIM thicknesses.

### 2.2. Preparation of PIMs

Membranes were prepared dissolving 30 mg of CTA, 75 mg of NPOE, and 30 mg of Aliquat 336 in 10 cm^3^ of CH_2_Cl_2_. This mixture was stirred for 1 h in a 50 cm^3^ beaker and then the solvent was allowed to evaporate for 24 h. After this time, the membranes were detached from the beakers and their diameter and thickness were measured with a Fowler IP54 micrometer. An average weight of (135.86 ± 2.05) mg, an average thickness of (95 ± 15) µm, and an average diameter of (3.00 ± 0.02) cm were determined (n = 54). The PIM thickness is almost double that previously reported in a similar sensor [30] to enhance the detectability of the analyte. 

### 2.3. Liquid-Solid Extraction

The obtained membranes were placed in a 50 cm^3^ polypropylene falcon tube together with 30 cm^3^ of Cr(VI) solutions at different concentrations. Stirring was performed for 20 min, taking a 1 cm^3^ aliquot every 2 min. At the end, the membranes were removed from the aqueous phase and the aliquots were brought to a volume of 2 cm^3^ to be able to measure their concentration by FAAS. Experiments were performed on a duplicate basis with an average RSD of 5%.

### 2.4. Metal Quantification

The absorbances corresponding to the samples and standards were measured by FAAS according to the conditions established by the manufacturer (354.7 nm wavelength (λ), 7 nm slit, with an air-acetylene flame (99.6%, Praxair, Danbury, CT, USA and an oil-free GS-003—Air compressor, PG Instruments, Loughborough, Leicestershire, UK), from 0 to 7 mg/dm^3^, using a hollow cathode lamp, and sensitivity check of 5 mg/dm^3^). The calibration curves were performed at each of the experimental conditions employed, mismatching the standards and experimental samples.

### 2.5. Measuring Infrared Spectra

The IR spectra were obtained by direct analysis of the PIMs in the transmission mode for the quantitative analysis of chromium in the mid-infrared region. The PIM was mounted on the transmission accessory of the equipment after sandwiching the membrane between two Petri dishes to avoid wrinkles and movement. The spectrum of each sample was acquired in the 400–5000 cm^−1^ region in triplicate with 30 scans per measurement to reduce the within-sample variation, which was found to be, on average, 1% RSD, and the mean value of the spectra was used in further data treatment. This procedure ensures the reduction in sample differences due to random variations in experimental conditions (e.g., aqueous volume, PIM contact area, PIM composition, etc.). 

### 2.6. Development of the Chemometric Model

The multivariate calibration method was built from the information provided by the IR spectra of the PIMs with a set of 54 samples covering 27 different concentrations each by duplicate with an interval ranging from 1.92 × 10^−7^ to 1.92 × 10^−6^ mol/dm^3^ (10–100 ppb). The spectral range used for quantitative analysis initially was 400–5000 cm^−1^, so the experimental matrix consisted of 54 rows corresponding to the samples and 4601 wavelengths. Further cross-validation was performed in the calibration set using Venetian blinds with 3 data splits and 2 samples per blind (thickness). A test set consisting of 10 samples acquired as the calibration test, but independently, had dimensions of 10 × 4601. Metal concentrations given along the work refer to the initial concentrations of the metal in the solution. Data processing consisted of baseline correction (automatic weighted least squares, order one) and mean-centering. The PLS-Toolbox 9.0 software (Eigenvector Research, Inc. Wenatchee, WA, USA) was employed for all chemometric analyses. An in-house-made MATHLAB 9.7 R2019b (Natick, Apple Hill Campus, MA, USA) program was used for the evaluation of the FOM.

## 3. Results and Discussion

### 3.1. PIM Composition

The composition of the PIMs used in this work was that reported by Kozlowski [20], substituting the plasticizer o-nitrophenyl pentyl ether for NPOE. With this membrane composition, the extraction experiments carried out at pH 6 in a 0.01 mol/dm^3^ MES buffer solution for 2 h with an initial metal concentration of 1.35 × 10^−4^ mol/dm^3^ (7 ppm) showed 90.2% extraction of the metal. This result agrees with the expectations from SX experiments [19]. Consequently, due to the high extraction percentage, it was decided to keep such proportions of the polymer (22.2% ± 0.7) *w*/*w*%, plasticizer (54.4 ± 1.3) *w*/*w*%, and extractant (23.4 ± 1.5) *w*/*w*%.

### 3.2. Optimization of the Extraction Time 

**Figure 1:**
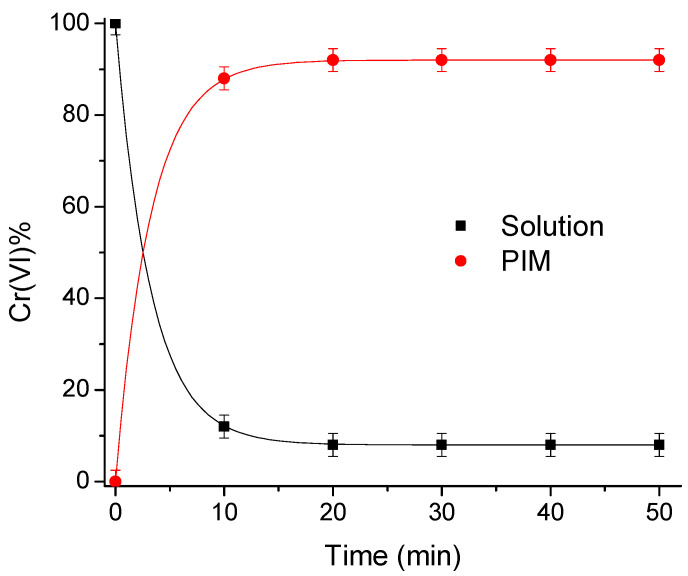
Cr(VI) extraction profile as a function of time (pH 6 in a 0.01 mol/dm^3^ MES buffer solution, [Cr(VI)]_0_ = 1.35 × 10^−4^ mol/dm^3^).

The extraction time necessary to reach the equilibrium was determined with a solution of 1.35 × 10^−4^ mol/dm^3^ Cr(VI), taking aliquots each 10 min for a total of 50 min. As observed in Figure 1, the concentration of the metal in the medium decreases until it reaches equilibrium, while the concentration of chromium in the membrane increases; thus, it can be concluded that the time required for the extraction reaction to reach equilibrium was 20 min. Consequently, further extraction experiments were carried out for 20 min of agitation at pH 6 with 0.01 mol/dm^3^ MES buffer to obtain the maximum extraction percentages.

### 3.3. Influence of pH

**Figure 2:**
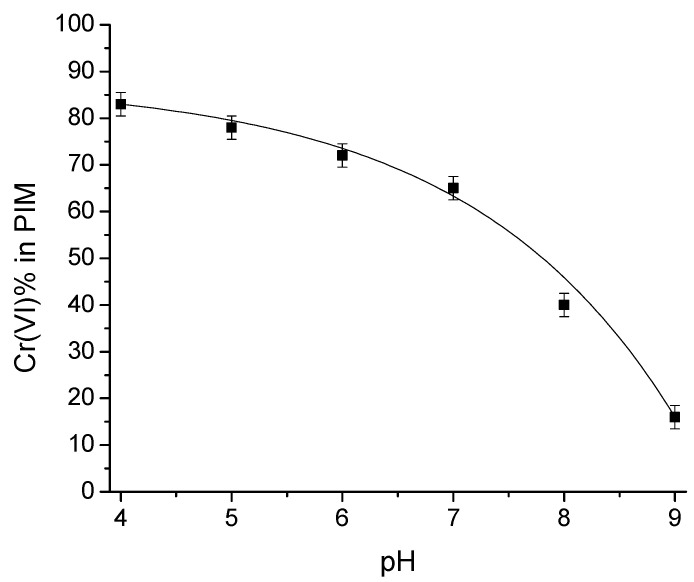
Cr(VI) extraction profile as a function of pH of the aqueous sample. [Cr(VI)]_0_ = 6.73 × 10^−4^ mol/dm^3^.

Knowing the time required to bring the extraction reaction to its equilibrium state, the influence of pH variations on metal extraction was studied. A concentration of 6.73 × 10^−4^ mol/dm^3^ (35 ppm) was used in the pH range 4 to 9, as this is usually the range found in natural waters [35]. In the experiments carried out at pH 4 and 5, a 0.01 mol/dm^3^ acetate/acetic acid buffer solution was used. For pH 6 and 7, a 0.01 mol/dm^3^ MES buffer was employed, and finally, for pH 8 and 9, a 0.01 mol/dm^3^ TRIS buffer was used. From Figure 2, it can be observed that chromium extraction decreases as pH increases in such a form that at pH 9, the extraction is less than 20%; however, at pH 4, the extraction exceeds 80%. This behavior is mainly due to the change in metal speciation (from HCrO_4_^−^ to CrO_4_^2−^) with the increase in pH of the solution, leading to a modification in the stoichiometry of the extraction reaction to neutralize the charge in the extracted metal anion, consistent with what is observed in liquid–liquid extraction [36]. This means that the form in which Cr(VI) is present in the system determines its extraction behavior. It is also clear from the graph that variations in pH values strongly affect E%, with the 4–7 interval being where changes of approximately 20% in E% are noticed. Considering that the PIM system will not be employed as a recovery method for the analyte, but for quantitative analysis, this implies that a constant pH value within this range can also be used with allowable results because E% remains constant and high enough to permit reproducible adsorption of the metal in the PIM. Therefore, even though the highest percentage of extraction was obtained at low pH, further experiments were performed at pH 6 since natural waters mostly register a pH around this value. 

### 3.4. Influence of the Initial Metal Concentration 

#### 3.4.1. Adsorption Isotherm

**Figure 3:**
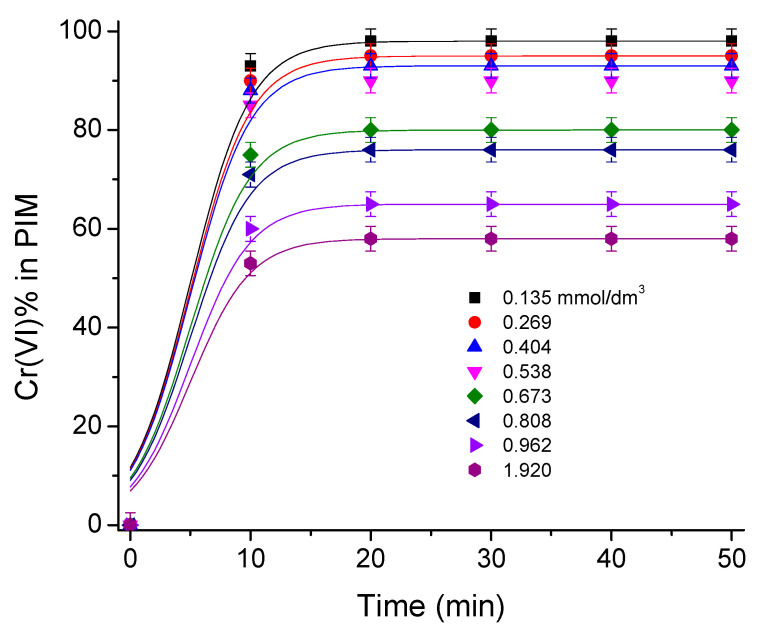
Cr(VI) extraction profile for different initial concentrations of metal at pH 6.

Under the extraction conditions established, as mentioned, the effect of the initial metal concentration was studied. This study was performed using initial metal concentrations from 1.35 × 10^−4^ mol/dm^3^ (7 ppm) to 1.93 × 10^−3^ mol/dm^3^ (100 ppm). From Figure 3, it is seen that the extraction with high initial metal concentrations is less effective than the extraction with low metal concentrations, i.e., the lower the initial metal concentration the more effective the extraction. It is also observed that the time to rich equilibrium depends on the initial concentration of the metal; however, above 15 min, all systems reach such condition.

**Figure 4:**
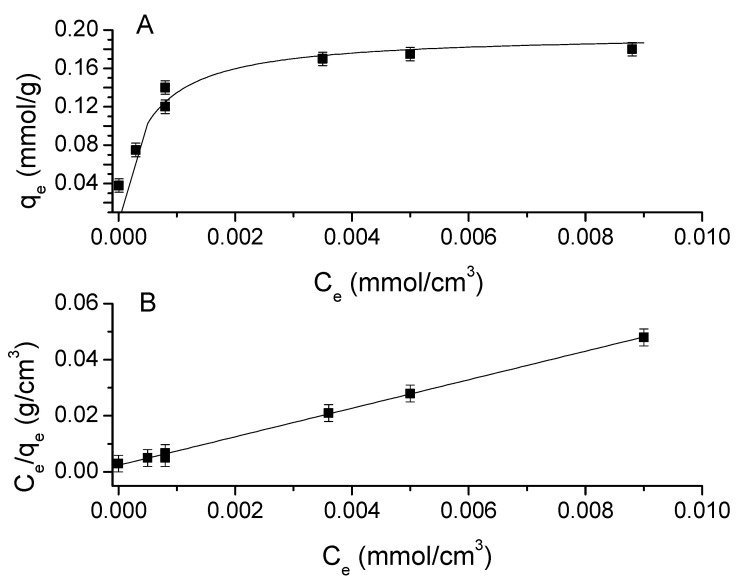
(**A**) Cr(VI) adsorption isotherm at pH 6. (**B**) Linearized form of the Cr(VI) Langmuir adsorption isotherm.

It is known that there is a quantity of metal that, when extracted, may saturate the active sites of the membrane, preventing it from continuing to extract the metal ion, meaning that the adsorption capacities depend on the different initial concentrations. In general, an adsorption isotherm is a curve that describes the phenomenon that governs the mobility of a substance from an aqueous medium to a solid phase at a constant temperature. Commonly, the mathematical correlation of this phenomenon is expressed graphically using the loading of the analyte in the solid phase (*q_e_*) as a function of the residual equilibrium concentration in the aqueous phase (*C_e_*) [37]. Over the years, a wide variety of adsorption isotherm models have been developed to explain the kinetics, thermodynamics, and potential of this phenomenon [38]. The adsorption isotherm for the Cr(VI)/PIM is shown in Figure 4A. 

From this, a Langmuir-type behavior is inferred. This model assumes monolayer analyte adsorption that occurs on localized sites that are equivalent without considering steric hindrance or adjacent interactions between adsorbed molecules, and it is described by the following equation [39]:

(1) $$q_{e}=\frac{q_{max}K_{L}C_{e}}{1+K_{L}\;C_{e}}$$

where *C_e_* is the solute concentration at equilibrium, *q_e_* is the amount of solute adsorbed at equilibrium, *q_max_* is the maximum loading capacity, and *K_L_* is Langmuir’s constant. The previous equation can be linearized as:

(2) $$\frac{C_{e}}{q_{e}}=\frac{C_{e}}{q_{max}}+\frac{1}{q_{max}K_{L}}$$

So when plotting *C_e_*/*q_e_* as a function of *C_e_*, *q_max_* and *K_L_* can be obtained from the abscissa and the slope of the line, respectively. Another parameter associated with the Langmuir adsorption model is the separation factor (*R_L_*) defined by [40]:

(3) $$R_{L}=\frac{1}{1+K_{L}\;C_{0}}$$

which indicates whether the nature of the adsorption is favorable or not (favorable (0 < *R_L_* < 1), linear (*R_L_* = 1), unfavorable (*R_L_* > 1), or irreversible (*R_L_* = 0)).

The linearized form of the adsorption isotherm is shown in Figure 4B. A determination coefficient of 0.999 indicates a good fit of the data to the Langmuir-type model. The parameters of the model are *K_L_* = 2199 cm^3^/mmol and *q_max_* = 0.188 mmol/g. In Figure 4A, the points represent the data and the line the fitting to the model showing good agreement. Table 1 provides the parameters and proof of adequacy of the model. Furthermore, the *R_L_* values at the different initial concentrations are shown in Table 2. The 0 < *R_L_* < 1 values indicate that metal adsorption is favorable for this system [40].

#### 3.4.2. Distribution Quotient

**Figure 5:**
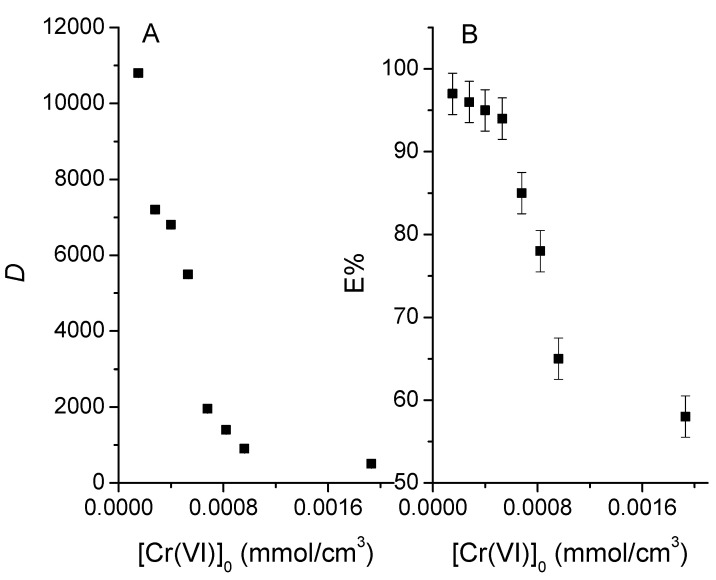
Variation of the distribution quotient of Cr(VI) (**A**) and the extraction percentage (**B**) as a function of the initial concentration of the metal.

The distribution quotient (*D*) is defined as the ratio of the sum of the concentrations of all the chemical forms of the compound in the aqueous phase and in the organic phase according to:

(4) $$D=\frac{\bar{\left[\mathrm{Cr}\left(\mathrm{VI}\right)\right]}}{\left[\mathrm{Cr}\left(\mathrm{VI}\right)\right]}$$

where the bar stands for the membrane phase. Figure 5A shows *D* as a function of the initial concentration of chromium from 1.35 × 10^−4^ to 1.93 × 10^−3^ mol/dm^3^. At low metal concentrations, *D* is ca10,000 units, which indicates a great affinity of the metal towards the membrane; on the other hand, a decay in its value at high concentrations is indicative of lower affinity, likely due to the saturation of the active sites, i.e., the extraction will be more effective at low metal concentrations. In general, the distribution into the organic phase of a metal complex increases with increasing temperature for complexes with significant hydrophobic character. The introduction of a complex into the organic phase involves several processes that can be associated with important changes in enthalpy (solvation processes) and entropy (solvent orientation and restructuring), leading to considerable temperature effects. In addition, to maintain electroneutrality and solute uphill pumping, the extraction systems require a coupling ion to be counter-transported along with the solute ion. Because the coupling ion must also cross the organic phase, it is bound to influence extraction efficiency [41].

#### 3.4.3. Extraction Percent

In Figure 5B, the extraction percentages are presented for D values. However, if the volumes of the phases are considered, *E*%, may also be defined as:

(5) $$E\%=\frac{\bar{mmol_{Cr}}}{mmol_{Cr\;0}}\times 100\%$$

At concentrations lower than 5 × 10^−4^ mol/dm^3^, *E%* is quantitatively high, with a value of 97.31%, and remains practically constant for the first 4 points. On the other hand, at higher concentrations, it decays, because of the occupation of the available sites on the membrane surface. From the point of view of quantitative analysis, initial concentrations lower than 5.5 × 10^−4^ mol/dm^3^ will be preferred to have constant extraction percentages, independent of the initial Cr(VI) content. 

#### 3.4.4. Enrichment Factor

The enrichment factor is a parameter that indicates how many times the metal is more concentrated in the membrane than in the solution, and is defined by [30]:

(6) $$E=\frac{\bar{\left[\mathrm{Cr}\left(\mathrm{VI}\right)\right]}}{{\left[\mathrm{Cr}\left(\mathrm{VI}\right)\right]}_{0}\;\;}$$

**Figure 6:**
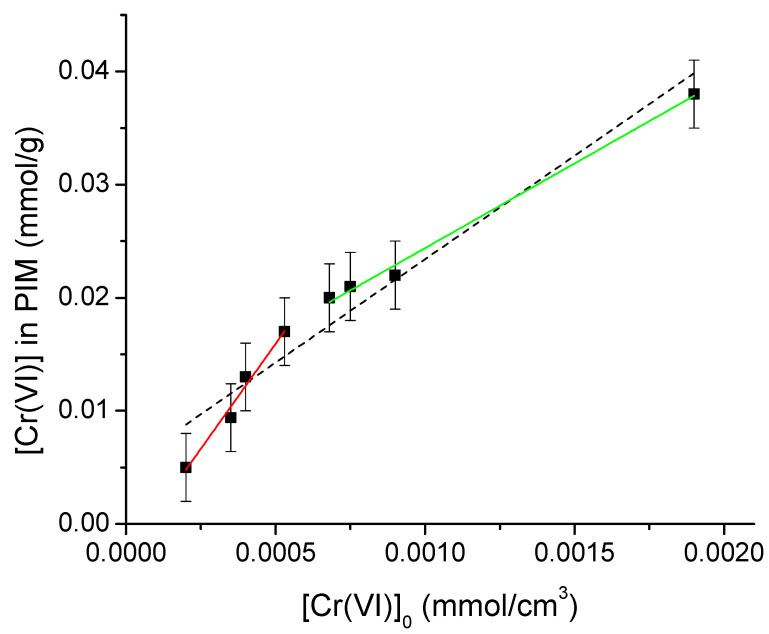
Determination of the enrichment factor, *E*, at different initial Cr(VI) concentrations, considering all points (black dotted line, slope = 18.39), first 4 points at low concentrations (red line, slope = 33.30), and last four points at high concentrations (green line, slope = 15.09).

*E* was evaluated within the range 1.35 × 10^−4^–1.95 × 10^−4^ mol/dm^3^ (Figure 6). From the slope of the graph, *E* ≈ 18. However, the graph clearly shows two trends according to the concentration range below and above 6 × 10^−4^ mol/dm^3^ (Figure 6). Dividing the values according to this point, the highest enrichment factor is obtained at low concentrations, with *E* ≈ 33 where the extraction percentage is constant and practically independent of the initial chromium concentration. On the other hand, the enrichment factor at high concentrations corresponds to *E* ≈ 15, with the extraction percentage depending on the initial chromium concentration. This observation highlights the need to work in the lower range of concentrations to obtain better results, since the higher the metal is loaded in the PIM the more sensitive the analytical method. The breakpoint noted in Figure 6 is related to the saturation of the active sites of the PIM, as previously discussed; where they are limited, their fulfillment is dependent on the initial metal concentration due to the competition from them established by the ions in solution. In contrast, when they are not limited, this competition is reduced due to the excess of sites available.

### 3.5. Determination of the Extraction Equilibrium

For this determination, the concentration of Aliquat 336 in the membrane was varied, keeping the amount of CTA and NPOE constant. The mass values of the Aliquat 336 used were 1.5, 5.1, 9, 11.1, 12, 15, 22.5, and 30 mg. According to Kebiche-Senhadji [23], extraction occurs by the following reaction:

(7) $$H_{i}CrO_{4}^{n-}+n\bar{L^{+}Cl^{-}}\to \bar{H_{i}CrO_{4}^{n-}L_{n}^{+}}+nCl^{-}$$

where $\bar{L^{+}Cl^{-}}$ stands for the extractant and $\bar{H_{i}CrO_{4}^{n-}L_{n}^{+}}$ for the extracted species in the PIM phase. The extraction constant, *K_ext_*_,_ is defined by:

(8) $$K_{ext}=\frac{\bar{\left[H_{i}CrO_{4}^{n-}L_{n}^{+}\right]}\;{\left[Cl^{-}\right]}^{n}}{[H_{i}CrO_{4}^{n-}]\;{\left[\bar{L^{+}Cl^{-}}\right]}^{n}}$$

Considering the definition of *D*, *K_ext_* can be rewritten as follows:

(9) $$K_{ext}=\frac{D\;{\left[Cl^{-}\right]}^{n}}{{\left[\bar{L^{+}Cl^{-}}\right]}^{n}}$$

Taking logarithms to both sides of the equation and rearranging:

(10) $$logD=logK_{ext}+nlog\left[\bar{L^{+}Cl^{-}}\right]-nlog[Cl^{-}]$$

**Figure 7:**
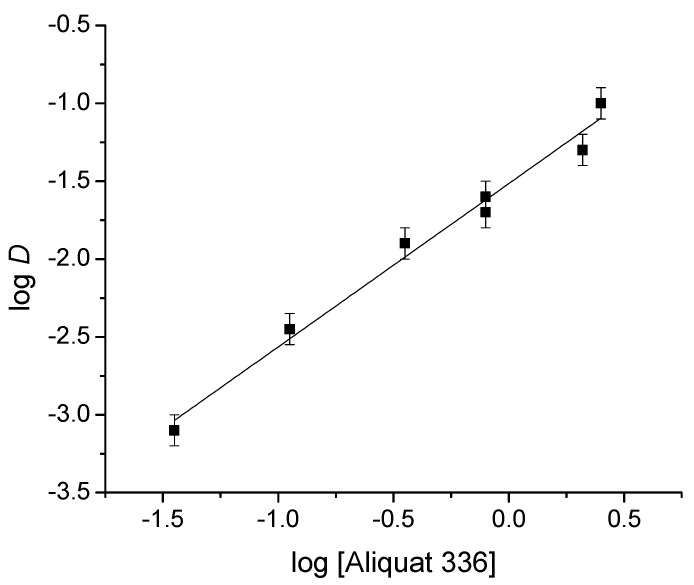
Determination of the stoichiometric ratio of the extracted Aliquat 336–Cr(VI) complex.

Figure 7 shows *logD* as a function of the unreacted log[Aliquat 336]; a linear relationship with a determination coefficient of 0.9902 was obtained. The slope value of 1.16 indicates that the Cr(VI): Aliquat 336 ratio is 1:1, so the final extraction reaction becomes:

(11) $$HCrO_{4}^{-}+\bar{L^{+}Cl^{-}}\to \bar{HCrO_{4}^{-}L^{+}}+Cl^{-}$$

This result is consistent with that reported in the literature, where the same stoichiometry was observed in SX [42].

### 3.6. Chemometric Analyses

To meet the chromium determination requirements in aqueous media [3,4,6] further experiments were performed at a lower concentration range (10–100 ppb) where, according to the previous discussions, the PIM system will perform the best.

#### 3.6.1. PCA

**Figure 8:**
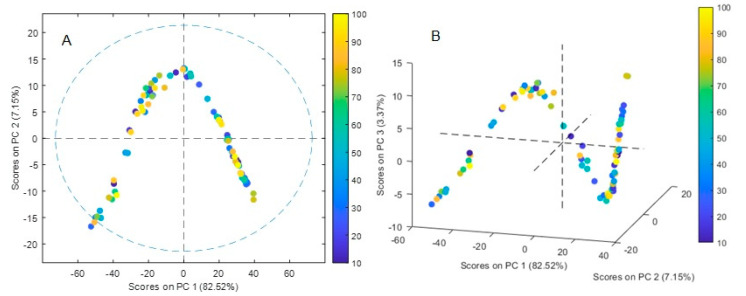
Score plot of the PCA analysis. The points are colored according to the initial Cr(VI) concentrations in aqueous solution. (**A**) PC1 vs. PC2, (**B**) PC1 vs. PC2 vs. PC3.

The sample score plots of the PCA analysis are shown in Figure 8A,B. A five-component model accounted for 95.38% of the variance in the X-block with 82.5% accounting exclusively for PC1. The *RMSEC* and *RMSECV* values were 0.10036 and 0.126592, respectively. A distinctive V-shape pattern observed in structured dependent data was identified in the plot PC1 vs. PC2 (Figure 8A). In gene population studies, PCA plots often appear triangular due to the underlying genetic population structure and the way genetic variation is distributed across individuals, as individuals with similar genetic ancestry tend to cluster together, at the three corners of the triangle, which represent different groups of individuals with unique genetic backgrounds [43,44]. This pattern also occurs in chemistry mixture problems with the three vertices being samples that contain a single component each, the samples falling on a line between two vertices being binary mixtures of the three analytes, and the remaining points being ternary mixtures of the three analytes [45]. Similar behavior has also been observed in optodes with 1-(2-pyridylazo)-2-naphthol as a chromophore where the proportions of the analytes (Cu(II), Zn(II), and Pb(II)) were systematically varied giving rise to mixtures of colors in the PIM [46]. However, in the present case, the pattern seems to be more complex as observed when analyzing the contribution of the first three PCs (Figure 8B), where a progressive change in the spectra apparently not related to the concentration of the analyte is observed. Although a complete understanding of factors giving rise to the observed shape is outside the present work, it can be related to dependent structured modifications in the PIM medium leading to non-linearities of the system, as the polar nature of water molecules adsorbed can induce local electric dipoles resulting in an increase in the effective polarizability of the material, affecting the penetration depth of the evanescent wave into the sample.

#### 3.6.2. Selection of the Spectral Wavelength Range

**Figure 9:**
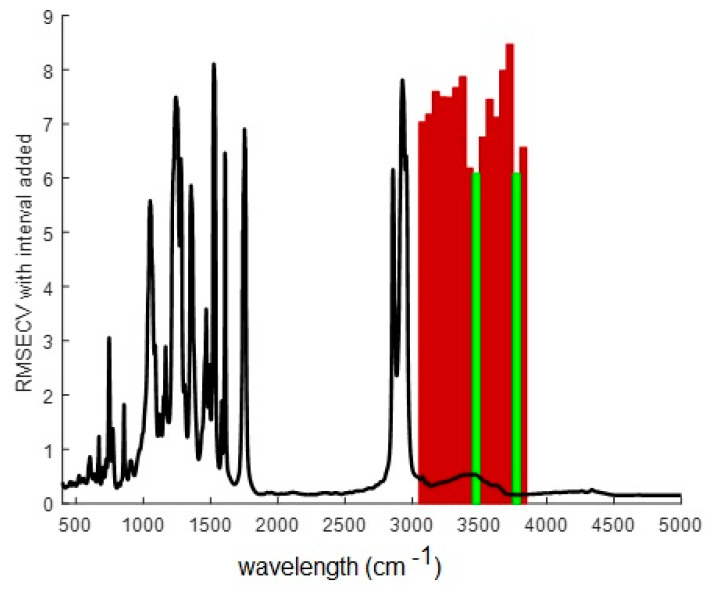
Forward iPLS results on the FTIR mean spectrum of the samples (black) showing the regions where minimum values of *RMSECV* were attained (red) and the optimal ones (green).

Preliminary full-spectrum PLS modeling did not give satisfactory results, as a 10 latent variable model gave *RMSEC* of 8.41851, *RMSECV* of 36.9153, Bias of 2.84217 × 10^−14^, CV Bias of 0.208479, R^2^ Cal of 0.905564, and R^2^ CV of 0.000624484, clearly showing model overfitting. This result is somehow expected from the previous PCA analysis where no evident trend with Cr(VI) concentrations was observed. Furthermore, no improvement was observed by changing the preprocessing method or the number of latent variables. However, high improvement was attained when some regions of the spectra were discarded from the calculations, so the forward interval PLS algorithm (iPLS), a variable selection method, was implemented. iPLS selects a subset of variables, which will give superior prediction compared to using all the variables in the dataset. It performs a sequential, exhaustive search for the best variable or combination of variables [47]. Figure 9 shows the obtained results. It was observed that when wavelengths in the 3050–3890 cm^−1^ region were considered (in red color in Figure 9), the best predictions were attained, with a minimum *RMSECV* value when bands in the ranges of 3451–3500 cm^−1^ and 3751–3800 cm^−1^ were chosen (in green in Figure 9). 

The extraction mechanism of Cr(VI) with Aliquat 336 involves ion-pair formation. Aliquat 336, being a cationic extractant, can form an ion pair with the hydrogen chromate ion through electrostatic interactions. The broad band in the FTIR spectra with the maximum located near 3500 cm^−1^ can be attributed both to hydroxyl stretching in CTA [47] and to stretching vibrations of adsorbed water in Aliquat 336 [22,28,48,49]. Fontàs et al. have found that modifications in the surface composition of water-equilibrated Aliquat 336-PIMs may be associated with the solubilization of the extractant in the water solution, which, therefore, may affect the reactivity of the membrane’s surface, but not the bulk properties of the membrane itself [50]. Such Aliquat 336 solubilized molecules may form micelles at the PIM [34], containing hydrated nonpolar cavities, such that the Me_3_N^+^ headgroup in Aliquat 336 and the HCrO_4_^−^ anion likely form solvent-separated ion pairs at the micelle surface [51]. This explains why this IR region performs the best in the developed method and can also be the reason for similar behavior, in which changes in such a band range can be noticed after the adsorption of Cr(VI) in an Aliquat 336 Dowex 1 × 8 impregnated resin [52] and the band shifting in the -OH stretching region reported for a CTA/1,5-diphenylcarbazide/Aliquat 336 optode [14].

#### 3.6.3. PLS Modeling

Hence, the final PLS model included the 100 wavelengths selected by iPLS and 10 latent variables according to the *RMSEC* and *RMSECV* values obtained using the calibration and cross-validation results. In Table 3, the percent of variance accounting for the different latent variables is shown. With the 10 latent variables, the variance captured by the model in the X-block reaches 99.99% while that in the y-block (Cr(VI) concentrations) is 97.17%. 

**Figure 10:**
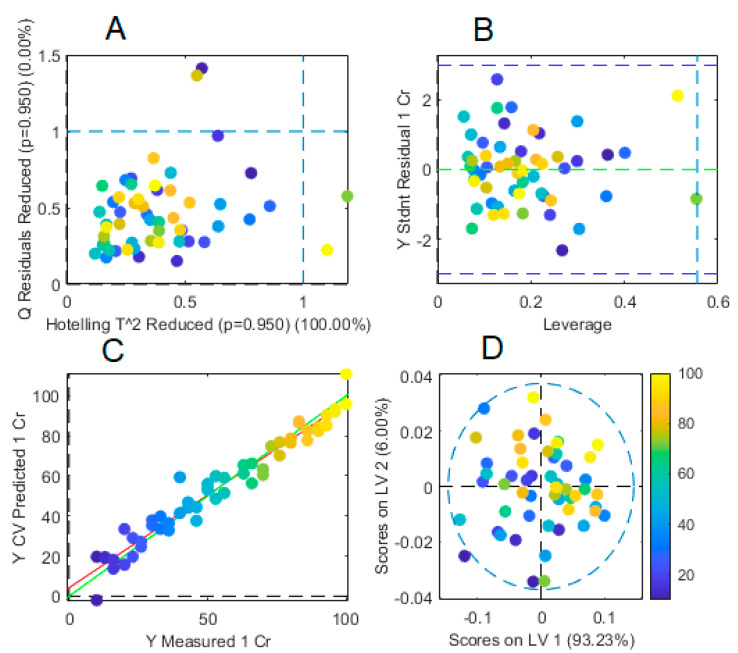
Summary of the results of the final PLS regression model. (**A**,**B**) Outliers plot, (**C**) parity plot, (**D**) plot of the first two latent variables showing the Hotelling T^2^ ellipse.

A summary of this final model is graphically presented in Figure 10A–D. No important outliers were detected in the data (Figure 10A,B) and the first two latent variables accounted for 99.24% of the variance in the X-block with almost all data being inside the Hotelling T^2^ ellipse (Figure 10D). Interestingly, the score plot did not show the complex pattern previously observed in PCA analysis this time, indicating efficient removal of variance not related to Cr(VI) concentrations modification, which this time moves sequentially from the third to the first quadrant in the plot as concentration increases (Figure 10D). The parity plot, i.e., measured vs. predicted concentrations (Figure 10C), showed excellent parameter values with *RMSEC* of 3.73115, *RMSECV* of 6.82685, Bias of −1.91847 × 10^−13^, CV Bias of 0.185947, R^2^ Cal of 0.98145, and R^2^ CV of 0.940902. 

### 3.7. Model Validation and Application

#### 3.7.1. Figures of Merit (FOM) [53,54,55]

When an analytical method is proposed, it is necessary to investigate whether it will perform adequately under the conditions where it will be further applied. This procedure is referred to as the validation of the method and it is performed through the determination of the FOM. These are very important parameters in characterizing, comparing, and developing new multivariate methods. Many of these figures are closely related to the net analyte signal (*NAS*) concept, as presented by Lorber [56] and defined by:

(12) $$NAS_{i}=\left(x_{i}\cdot b\right)\cdot {\left(b^{T}\cdot b\right)}^{-1}\cdot b^{T}$$

where *x_i_* is a sample spectrum after preprocessing and *b* is a column vector of the PLS regression coefficients. The concept of *NAS* arises naturally in multivariate calibration from the fact that a predicted sample spectrum can have various contributions from all the sample components. Therefore, it is logical to decompose the spectrum into two orthogonal parts: A part that can be exclusively assigned to the analyte of interest (*NAS*) and the remaining part that contains the contribution, possibly variable, of other components [57]. This decomposition is carried out by the regression algorithms in such a way that the *NAS* is proportional to the concentration of the analyte of interest. Since the *NAS* is the only part of the spectrum that is used for prediction, no information is lost by transforming the *NAS* vector into a scalar. The natural choice is to take the Euclidean norm, i.e., its length so that the scalar *NAS* is obtained as r* = ||r*||, where *r* is an arbitrary vector. Using the *NAS*, a multivariate calibration model can be represented on a pseudo-univariate plot, with this representation being exact, not approximate. In other words, the calibration curve is obtained by replacing the measured instrumental signal used in univariate calibration (e.g., absorbance at a single wavelength) with the NAS (r*) allowing a simpler interpretation of the response signal [53].

##### Accuracy (*RMSE*)

This parameter indicates the closeness of the relationship between the reference value and that found by the model. In multivariate calibration, it is usually expressed as the root mean square error of calibration (*RMSEC*), root mean square error of cross-validation (*RMSECV*), and root mean square error of prediction (*RMSEP*), according to the following equation:

(13) $$RMSE=\sqrt{\frac{\sum_{i=1}^{n}{\left(y_{i}-\hat{y_{i}}\right)}^{2}}{n}}$$

where $y_{i}$ and $\hat{y_{i}}$ are the estimated value of the model and the reference value of sample *i*, respectively, and *n* is the number of samples. The estimated value varied according to what is measured, the calibration (*RMSEC*), cross-validation (*RMSECV*), or the test (*RMSEP*) results.

##### Selectivity (*sel*)

This indicates the part of the total signal that is not lost due to spectral overlapping and can be defined in the multivariate context through the *NAS* calculation:

(14) $$sel=\frac{\left|\left|s_{k}^{*}\right|\right|}{\left||s_{k}|\right|}$$

where $\left||s_{k}|\right|$ stands for the norm of the sensitivity coefficients of the spectra containing the analyte *k* at unit concentration, and $\left|\left|s_{k}^{*}\right|\right|$ for that corresponding to its *NAS*.

##### Sensitivity (*sen*)

Sensitivity measures the changes in the response, as a function of the concentration of a particular analyte, and is given by the following equation:

(15) $$sen=||s_{k}^{*}||$$

However, it can also be calculated without the use of NAS theory according to the equation:

(16) $$sen=\frac{1}{\left|\left|b\right|\right|}$$

A more useful FOM is the analytical sensitivity (γ), which is defined, by analogy with univariate calibration, as the ratio between *sen* and the instrumental noise (*δx*). The inverse of γ (γ^−1^) provides an estimation of the minimum concentration difference that is discernible by the analytical method considering the random experimental noise as the only source of error, regardless of the specific technique employed [57].

##### Limit of Detection (*LD*)

Following the IUPAC recommendations, *LD* can be defined as the minimum detectable value of the net signal for which the false negative (β) and false positive (α) probabilities are 0.05. *LD* can be calculated analogously, as for univariate calibration, according to the equation:

(17) $$LD=3.3\delta x\frac{1}{sen}$$

##### Limit of Quantitation (*LQ*)

Quantizability is generally expressed in terms of the signal or analyte concentration value that will produce estimates with a specified relative standard deviation, usually 10% RSD. Following the same assumptions described above, the quantitation limit in the multivariate calibration can be calculated by:

(18) $$LQ=10\delta x\frac{1}{sen}$$

The calculated FOM of the PLS model is given in Table 4. The absence of systematic errors in accuracy determination was verified with an *F*-test over the parity plot during cross-validation (Figure 10C). In the absence of errors, it is expected that the slope obtained from this graph is equal to 1 (*β*_1_ = 1) and the abscissa is 0 (*β*_0_ = 0) (null hypothesis). The alternative hypothesis states that at least one of these parameters is different from the expected value.

This simultaneous null hypothesis can be tested from [30,56]:

(19) $$F=\frac{{\left(\beta_{0}-b_{0}\right)}^{2}+2\bar{x}\left(\beta_{0}-b_{0}\right)\left(\beta_{1}-b_{1}\right)+\left(\sum x_{i}^{2}/n\right){\left(\beta_{1}-b_{1}\right)}^{2}}{2(S_{e}^{2}/n)}$$

where:

*β*_0_ = 0,
*β*_1_ = 1,
*b*_0_ = observed abscissa,
*b*_1_ = observed slope,
and $S_{e}=\sqrt{\frac{\sum {\left(y_{i}-{\hat{y}}_{i}\right)}^{2}}{n-2}}$.

The obtained *F*-value is then compared to an *F* distribution reference value with 2 and *n* − 2 degrees of freedom at the chosen significance level.

From Figure 10C, *b*_0_ = 4.22147, *b*_1_ = 0.924373, $\bar{x}=5$4.4814, *n* = 54, and *Se* = 5.916 giving *F* = 3.32. As this value is smaller than the tabulated F^0.05^ _2, 52_ = 3.96, the hypothesis of *β*_1_ = 1, *β*_0_ = 0 is accepted, i.e., no systematic bias is present.

The model presents a sensitivity value of 0.0015%, indicating that it is capable of distinguishing samples with concentration differences of 0.6 ppb. The selectivity value indicates that 1.5% of the information on the analyte contained in the sample is orthogonal to the interference space. In other words, the selectivity indicates that approximately 98.5% of the interfering analytical signals were removed during the NAS calculation. This result is expected due to the low concentration range of the analyte in relation to the other PIM components, meaning that only slight changes in the PIM spectra were related to Cr(VI) concentration variation. However, even such low variability was effectively accounted by the PLS algorithm. On the other hand, the well-probed chemical selectivity of Aliquat 336 for Cr(VI) over Co(II), Cd(II), Pb(II), Ni(II), Cu(II), Zn(II), and Fe(II) [23,27,28,58] favors the testing of the PIM on real samples.

Table 5 compares the results of the developed sensor to those reported in the literature for Cr(VI) analysis with comparable systems. Similar FOM with other Cr(VI) optical quantitation methods were found. However, as most of the works reported in Table 5 rely on the use 1,5-diphenylcarbazide as a chromophore, they are limited to work at low pH values, where the highest absorbance values are attained [13,14], with those methods then being not suitable for in-situ analysis of water samples. In comparison to some of them, the developed PIM sensor is, in many cases, much easier to implement as it requires few manipulations and a reduced number of chemical compounds.

#### 3.7.2. Application

The developed PLS method was applied to each of the test samples, and the *t*-value for the comparison between the measured (reference) and predicted (found) concentrations was calculated. The results are shown in Table 6. As the obtained *p*-value > 0.05, there were no significant differences between both quantities at the 95% confidence level. The *RMSEP* value given in the same table is close to the *RMSEC* and *RMSECV* values, meaning that model overfitting was prevented. An average recovery of 104.02 ± 4.12 (α = 0.05) was obtained.

## 4. Conclusions

Using infrared spectroscopy and multivariate statistical analysis, a MID-FTIR-PLS PIM-based sensor for the quantitative determination of Cr(VI) from an aqueous medium was developed with satisfactory results. Optimized conditions for the system consisted of (22.2 ± 0.7) *w*/*w*%, CTA, (54.4 ± 1.3) *w*/*w*% NPOE and (23.4 ± 1.5) *w*/*w*% Aliquat 336, pH 6, and 20 min of extraction time, although pH values in the range of 4–7 could also be used. It was observed that chromium extraction decreases as the pH increases, in such a form that at pH 9, the extraction is less than 20%. However, at pH 4, the extraction exceeds 80%; this behavior is mainly due to the change in metal speciation (from HCrO_4_^−^ to CrO_4_^2−^) with increasing pH of the solution. Cr(VI) adsorption followed a Langmuir-type isotherm with *K_L_* = 2199 cm^3^/mmol, *q_max_* = 0.188 mmol/g and 0 < *R_L_* < 1. At low metal concentrations, *D* is ca.10,000 units, which indicates a great affinity of the metal towards the membrane; on the other hand, a decay in its value at high concentrations is indicative of lower affinity, as also observed in the *E%* profile. The highest enrichment factor was obtained at low analyte concentrations, with *E* ≈ 33, where the extraction percentage is practically constant. On the other hand, the enrichment factor at high concentrations corresponds to *E* ≈ 15. Characterization of the extraction reaction indicated a 1:1 Cr(VI): Aliquat 336 ratio. The PCA analysis of the PIMs revealed a complex pattern, which was satisfactorily simplified and related to Cr(VI) concentrations through the use of a variable selection method (iPLS) in which bands in the ranges of 3451–3500 cm^−1^ and 3751–3800 cm^−1^ were chosen. The final PLS model including the 100 wavelengths selected by iPLS and 10 latent variables shows excellent FOM values. The developed PIM sensor is suitable for in situ analysis of aqueous samples, and it is easy to implement as it requires few manipulations and a reduced number of chemical compounds in comparison to other similar reported systems.

## Figures and Tables

**Table 1:** Values of the parameters and the fitting results of the Langmuir model equation.

Parameter	Value
*q_max_*	0.188 mmol/g
*K_L_*	2199 cm^3^/mmol
Reduced Chi-Sqr	0.02628
Adjusted R^2^	0.90717
ANOVA:	
Regression sum of squares	0.0247
Residual sum of squares	0.0024
Regression mean square	0.0123
Residual mean square	0.00048
*F*-value	25.6361
*p*-value	0.0023

**Table 2:** Values of the *R_L_* parameter of the Langmuir model at different initial Cr(VI) concentrations.

${\left[Cr\left(VI\right)\right]}_{0}(\mathrm{mg}/{\mathrm{dm}}^{3})$	*R_L_*
6.81	0.0064
14.53	0.0031
20.65	0.0021
27.10	0.0016
36.74	0.0012
40.73	0.0010
48.70	0.0009

**Table 3:** Percent of variance captured by the PLS Regression Model.

	X-Block		y-Block	
Component	This Component	Total	This Component	Total
1	93.23	93.23	10.71	10.71
2	6.00	99.24	14.71	25.41
3	0.48	99.72	51.55	76.96
4	0.23	99.95	7.35	84.31
5	0.03	99.98	3.30	87.62
6	0.01	99.99	7.49	95.10
7	0.00	99.99	2.07	97.17

**Table 4:** Analytical figures of merit for the final PLS regression model.

FOM		Results
Accuracy	*RMSEC*	3.73115
	*RMSECV*	6.82685
	*RMSEP*	3.3229
	Bias	−1.91847 × 10^−13^ (Cal)
		0.185947 (CV)
	R^2^	0.98145 (Cal)
		0.940902 (CV)
	Recovery%	104.02 ± 4.12 * (Test)
*sen*		0.00001547 ppb
γ		3.8 ppb
γ^−1^		0.6 ppb^−1^
*sel*		0.0155
Linear range		5.8–100 ppb
*LD*		1.9 ppb
*LQ*		5.8 ppb

* 95% confidence level.

**Table 5:** Comparison of some representative works for Cr(VI) quantitation reported in the literature *.

Detection Method	Carrier/Chromophore	Linear Range	pH	*LD*	*LQ*	Reference
Colorimetry	2-hydroxy, 3-methoxy benzaldehyde thiosemicarbazone	0.260–2.60 μg/cm^3^	6	0.014 μg/cm^3^	0.041 μg/cm^3^	[59]
Colorimetry	1,5-diphenylcarbazide	0.03–3 μg/cm^3^	2.2	0.023 μg/cm^3^	0.076 μg/cm^3^	[60]
Colorimetry	diazonium salt and citrazinic acid	0.2–1.5 μg/cm^3^	Alkaline medium	0.04 μg/cm^3^		[61]
Rotational microfluidic paper-based device	1,5-diphenylcarbazide	0.5–10 μg/cm^3^	Very acidic	0.18 μg/cm^3^		[62]
Sol-gel monoliths	pyridine-functionalized sol-gel monoliths and diphenylcarbazide		1	about 0.010 μg/cm^3^		[12]
Optode	aliquat 336 and 1,5-diphenylcarbazide	0.020–0.397 μg/cm^3^	3	0.011 μg/cm^3^	0.013 μg/cm^3^	[11]
Optode	1,5-diphenylcarbazide	0.0024–1 μg/cm^3^	1	0.0007 μg/cm^3^	0.0024 μg/cm^3^	[13]
Optode	aliquat 336 and 1,5-diphenylcarbazide	0.02–0.40 μg/cm^3^	3	0.0055 μg/cm^3^	0.0165 μg/cm^3^	[14]
FTIR optode	aliquat 336	0.0058–0.1 μg/cm^3^	6	0.0019 μg/cm^3^	0.0058 μg/cm^3^	This work

* Units of concentration have been standardized to facilitate the comparison of the different reported methods.

**Table 6:** Results of the analysis of the test samples by the final PLS regression model.

Sample	Measured (ppb)	Predicted (ppb)	Recovery%	*RMSEP*	*p*-Value
Test 1	13	13.99	107.66		
Test 2	20	23.57	117.86		
Test 3	36	37.25	103.47		
Test 4	40	42.32	105.81		
Test 5	63	63.23	100.37		
Test 6	73	75.85	103.90		
Test 7	80	77.89	97.48		
Test 8	86	89.07	103.57		
Test 9	90	90.90	101.01		
Test 10	100	99.00	99.00		
Average			104.02		
				3.3229	0.0611

## Data Availability

The data that support the findings of this study are available upon reasonable request.

## Abbreviations

Aliquat 336	Methyltrioctylammonium chloride
Cal	Calibration
CTA	Cellulose triacetate
CV	Cross-validation
*D*	Distribution coefficient
*E*	Enrichment factor
*E%*	Extraction percentage
FAAS	Flame atomic absorption spectroscopy
FOM	Figures of merit
FTIR	Fourier-transform infrared spectroscopy
iPLS	Interval PLS
IR	Infrared spectroscopy
ISOs	Ion-selective optodes
*K_ext_*	Extraction constant
*LD*	Limit of detection
*LQ*	Limit of quantitation
MES	2-(N-morpholino) ethanesulfonic acid
MID-FTIR	Mid Fourier transform infrared spectroscopy
*NAS*	Net analyte signal
NPOE	2-nitrophenyl octyl ether
PCA	Principal component analysis
PIM(s)	Polymer inclusion membrane(s)
PLS	Partial least squares
R^2^	Determination coefficient
*RMSEC*	Root mean square error of calibration
*RMSECV*	Root mean square error of cross-validation
*RMSEP*	Root mean square error of prediction
RSD	Relative standard deviation
*sel*	Selectivity
*sen*	Sensitivity
SIR	Solvent-impregnated resins
SLM	Supported liquid membranes
SX	Solvent extraction
TRIS	Tris(hydroxymethyl)aminomethane
VIS	Visible spectroscopy
